# Protective Effects of Human Liver Stem Cell-Derived Extracellular Vesicles in a Mouse Model of Hepatic Ischemia-Reperfusion Injury

**DOI:** 10.1007/s12015-020-10078-7

**Published:** 2020-12-02

**Authors:** Alberto Calleri, Dorotea Roggio, Victor Navarro-Tableros, Nicola De Stefano, Chiara Pasquino, Ezio David, Giada Frigatti, Federica Rigo, Federica Antico, Paola Caropreso, Damiano Patrono, Stefania Bruno, Renato Romagnoli

**Affiliations:** 1 General Surgery 2U, Liver Transplantation Center, AOU Città della Salute e della Scienza di Torino, University of Turin, Turin, Italy; 2 Scarl. - Molecular Biotechnology Center (MBC), 2i3T - Società per la gestione dell’incubatore di imprese e per il trasferimento tecnologico dell’Università degli Studi di Torino, Turin, Italy; 3 Department of Medical Sciences, University of Turin, Turin, Italy; 4 Pathology Unit, Molinette Hospital, AOU Città della Salute e della Scienza di Torino, Turin, Italy; 5 Forb – Fondazione per la Ricerca Biomedica, ONLUS - Molecular Biotechnology Center (MBC), Turin, Italy; 6 Clinical Biochemistry Laboratory, Molinette Hospital, AOU Città della Salute e della Scienza di Torino, Turin, Italy

**Keywords:** Ischemia-reperfusion, Hepatic inflammation, Adult stem cells, Microvesicles, Liver regeneration

## Abstract

Hepatic ischemia-reperfusion injury (IRI) is observed in liver transplantation and hepato-biliary surgery and is associated with an inflammatory response. Human liver stem cell-derived extracellular vesicles (HLSC-EV) have been demonstrated to reduce liver damage in different experimental settings by accelerating regeneration and by modulating inflammation. The aim of the present study was to investigate whether HLSC-EV may protect liver from IRI in a mouse experimental model. Segmental IRI was obtained by selective clamping of intrahepatic pedicles for 90 min followed by 6 h of reperfusion. HLSC-EV were administered intravenously at the end of the ischemic period and histopathological and biochemical alterations were evaluated in comparison with controls injected with vehicle alone. Intra liver localization of labeled HLSC-EV was assessed by in in vivo Imaging System (IVIS) and the internalization into hepatocytes was confirmed by fluorescence analyses. As compared to the control group, administration of 3 × 10^9^ particles (EV1 group) significantly reduced alanine aminotransferase (ALT) and lactate dehydrogenase (LDH) release, necrosis extension and cytokines expression (TNF-α, CCL-2 and CXCL-10). However, the administration of an increased dose of HLSC-EV (7.5 × 10^9^ particles, EV2 group) showed no significant improvement in respect to controls at enzyme and histology levels, despite a significantly lower cytokine expression. In conclusion, this study demonstrated that 3 × 10^9^ HLSC-EV were able to modulate hepatic IRI by preserving tissue integrity and by reducing transaminases release and inflammatory cytokines expression. By contrast, a higher dose was ineffective suggesting a restricted window of biological activity.

**Supplementary Information:**

The online version contains supplementary material available at 10.1007/s12015-020-10078-7.

**Figure:**
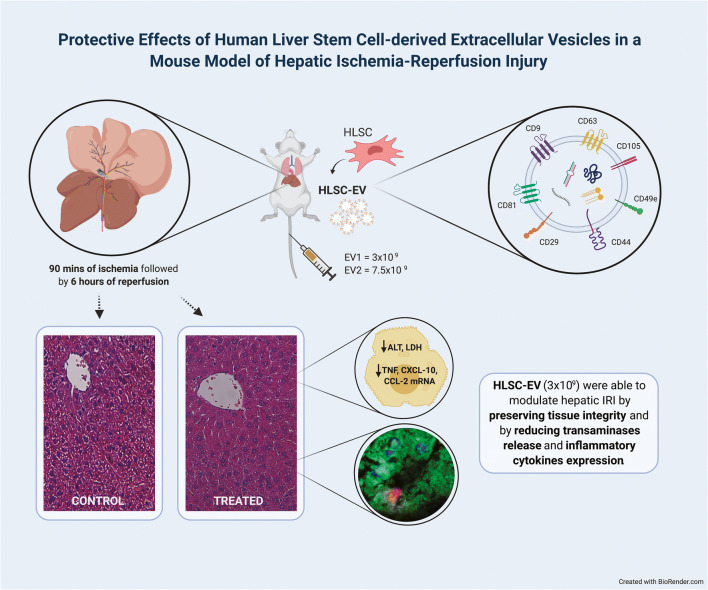
Graphical abstract

## Introduction

Hepatic ischemia-reperfusion injury (IRI) is an antigen-independent inflammatory response commonly observed when blood supply is restored after surgical procedures such as liver resection and transplantation. This phenomenon is initiated in Kupffer cells and hepatocytes by a burst in reactive oxygen species (ROS) production in mitochondria after organ reperfusion [1]. ROS production leads to hepatocyte and endothelial cell damage, promoting the recruitment of neutrophils and T-cells and starting an inflammatory cascade that eventually triggers apoptosis and necrosis [2–4]. In liver transplantation, IRI can cause early graft failure or dysfunction, and it is associated with a higher incidence of acute and chronic rejection [5].

Adult human liver stem-like cells (HLSC) were identified as a population of pluripotent resident liver cells expressing both markers characteristic of the mesenchymal lineage (CD29, CD44, CD73, CD90, CD105) and hepatic markers (albumin and alpha-fetoprotein), suggesting a partial hepatic commitment. [6] Moreover, these cells express vimentin, nestin, Musashi stem cell markers and nanog, SSEA4, pax2, and octa4 embryonic stem cell markers. [7, 8] HLSC were shown to localize within the injured tissue and to promote liver regeneration in a murine model of fulminant liver failure [7] and to increase kidney recovery in a murine model of acute kidney injury [8]. When seeded in acellular liver scaffold, HLSC were shown to differentiate into mature functional hepatocytes [9]. More recently, a phase 1 study demonstrated the safety of HLSC administration in infants with neonatal hyperammonemia [10]. Growing evidence supports the hypothesis that the biological effects of stem cells on neighboring cells are mediated by paracrine mechanisms related to the release of soluble factors and extracellular vesicles (EV) [11, 12]. EV are a heterogeneous population of cell-derived membrane vesicles originating from the endosomal compartment or from direct budding of plasma membrane, which are able to modulate phenotype and function of neighboring cells [13–15]. Several studies suggest that EV contribute to the regenerative effect of stem cells through a horizontal transfer of biological active proteins, lipids and specific subsets of messenger RNA and microRNA (miRNA) [16–19]. In particular, we demonstrated that EV derived from HLSC (HLSC-EV) were able to reduce apoptosis and to promote hepatocyte proliferation in a mouse model of partial hepatectomy [20]. More recently, we described the biological effects of HLSC-EV on livers perfused ex-vivo under hypoxic conditions [21]. Moreover, a protective role of HLSC-EV was demonstrated in a murine model of diet-induced non-alcoholic steatohepatitis (NASH) by showing anti-fibrotic and anti-inflammatory effects [19].

The regenerative properties of HLSC-EV have not been so far tested in a model of warm hepatic IRI. In liver resection, intermittent clamping of the hepatic pedicle (Pringle maneuver) is frequently employed to reduce blood loss during parenchymal transection, exposing liver cells to warm IRI and favoring post-hepatectomy liver failure. The latter is one of the main concerns after liver resection and a major determinant of postoperative morbidity and mortality.

The aim of the present study was to investigate whether HLSC-EV have a potential application in the setting of liver surgery by evaluating their effect in an experimental mouse model of warm hepatic IRI.

## Materials and Methods

### Animals

Animal studies were performed following a protocol approved by the Ethic Committee of the Italian Institute of Health (Istituto Superiore di Sanità, N.62/2016-PR). Male C57BL/6 mice at 8–10 weeks of age were used in all experiments and were housed in Molecular Biotechnology Center (Turin, Italy) animal facility under specific pathogen-free conditions, receiving human care according to the criteria of the National Institute of Health Guide for Care and Use of Laboratory Animals. Mice were maintained on a 12-h dark–light cycle and allowed free access to standard food and water. All experiments were conducted during the light cycle.

### Mouse Model of Liver IRI

Total anesthesia was induced through an intramuscular injection of tiletamine-zolazepam (Zoletil®) (0.2 mg/Kg) and xilazine (Rompun®) (16 mg/Kg). After a midline laparotomy, the falciform ligament was cut and the liver mobilized, allowing the exposure of the hepatic hilum. Vascular pedicles to the left lateral and median lobes (approximately 70% of total liver parenchyma) were clamped using an atraumatic clamp for 90 min. The non-ischemic lobes guaranteed a portocaval shunt avoiding intestinal congestion during the ischemic period (supplementary material 1). To prevent hypothermia, the abdomen was closed with a cutaneous running suture (silk 6/0) and the animal was kept warm under an infrared lamp. After 90 min of warm ischemia, the laparotomy was reopened and the clamp removed, allowing reperfusion of the whole liver. Immediately after reperfusion, HLSC-EV or vehicle (saline), according to the experimental group, were administered through the tail vein.

A total of 40 mice were used in the study. Animals were randomly shuffle ordered within four groups. From them, two mice were excluded from the analysis because one died by complications during anesthesia, while the second one was excluded because less than 70% of the liver parenchyma showed to be ischemic. Thereby, 38 mice, comparable in size and weight, were included in the analysis, and the groups were defined as follows: a) EV1 group (*n* = 10) received 3 × 10^9^ HLSC-EV diluted in 120 μl of saline; b) EV2 group (*n* = 9) received 7.5 × 10^9^ HLSC-EV diluted in 120 μl of saline; c) control group (n = 10) received 120 μl of saline; and d) sham operated group (n = 9).

All the animals from control, EV1 and EV2 groups underwent the laparotomy and clamping surgical procedures, followed by the intravenous injection (saline or HLSC-EV), while the sham group underwent the same surgical procedure except for the clamping and the intravenous injection.

All surgeries and intravenous injections were performed by the same operator. After being anesthetized, all the animals were sacrificed after 6 h post-reperfusion by exsanguination and cervical dislocation, then tissue samples were collected.

All the analyses included in our study (IVIS, biochemistry, histology and molecular biology) were blindly performed by different investigators.

### Isolation, Characterization and Culture of HLSC

The HLSC were isolated from human cryopreserved hepatocytes obtained from Lonza, Bioscience (Basel, Switzerland) as previously described [8]. The HLSC were cultured in a medium containing a 3:1 proportion of α-minimum essential medium and endothelial cell basal medium-1, supplemented with L-glutamine 2 mM, penicillin 100 U/mL, streptomycin 100 μg/mL and 10% fetal calf serum (α-MEM/EBM/FCS), and maintained in a humidified 5% CO_2_ incubator at 37°C [6]. HLSC at passages 5 to 8 were used in all the experiments.

HLSC were positive for CD73, CD90, CD105, CD29 and CD44 and negative for CD45, CD14, CD34, CD117 (c-kit) and CD133.

### Isolation and Characterization of HLSC-EV

The HLSC-EV were obtained as previously described [20]. Briefly, the HLSC were starved overnight in RPMI medium deprived of FCS at 37°C in a humidified incubator with 5% CO_2_. Viability of cells evaluated by trypan blue exclusion at the time of supernatant collection was >95%. Supernatants were collected, centrifuged for 30 min at 3000 g and submitted to microfiltration with 0.22-mm filters to remove cell debris and apoptotic bodies. Supernatants were then collected and ultracentrifuged at 100,000 g for 2 h at 4°C (Beckman Coulter Optima L-90 K, Fullerton, CA, USA). EV were collected and labelled with 1 μM of Dil dye and 1 μM of Did dye (1,1′-dioctadecyl-3,3,3′,3′- tetramethylindocarbocyanine perchlorate, Dil; 1,1′-dioctadecyl- 3,3,3′,3′-tetramethylindodicarbocyanine, 4-chlorobenzenesulfonate, Did; both from Molecular Probes Life Technology, New York, NY, USA), then washed in phosphate buffered saline (PBS) and ultracentrifugated for 1 h at 4°C [22]. The collected Dil-Did stained EV were used fresh or stored at −80°C after re-suspension in RPMI and 1% dimethyl sulfoxide. No differences in biological activity were observed between fresh and stored EV (data not shown). Quantification and size distribution of EV diluted (1:200) in sterile saline solution were performed by using NanoSight LM10 (NanoSight Ltd., Minton Park, UK) with the NTA 1.4 Analytical Software as previously described [23].

HLSC-EV were characterized by bead-based multiplex analysis by flow cytometry (MACSPlex Exosome Kit, human, Miltenyi Biotec) [24, 25]. Briefly, 1 × 10^9^ EV were diluted with MACSPlex buffer (MPB) to a final volume of 120 μL and loaded into a 1.5-mL tube. Thereafter, 15 μL MACSPlex Exosome Capture Beads (containing 39 different antibody-coated bead subsets) was added to each tube. To stain EV bound to beads, 5 μL of APC-conjugated anti-CD9, anti-CD63, and anti-CD81 detection antibodies were added to each tube and then incubated in an orbital shaker for 1 h at 450 rpm at room temperature in the dark. Beads were washed with 1 mL MPB and centrifuged at 3000 g for 5 min. A second step of washing with 1 mL MPB was performed by incubation in an orbital shaker at 450 rpm, in the dark for 15 min at room temperature and then submitted to centrifugation at 3000 g for 5 min. Flow-cytometric analysis was performed with a CytoFLEX instrument (Beckman Coulter, Brea, CA, USA) recording approximately 5000–8000 single-bead events per sample.

After background correction, the median fluorescence intensity (MFI) of all 39-capture beads subsets was recorded. All bead populations can be identified and gated based on their respective fluorescence intensity according to the manufacturer’s instructions.

For transmission electron microscopy analysis EV were placed on 200-mesh nickel formvar carbon-coated grids (Electron Microscopy Science, Hatfield, PA, USA) and after 20 min adhesion, followed by washing in PBS, EV were fixed with 2.5% glutaraldehyde containing 2% sucrose. After repeated washings in distilled water, the EV were negatively stained with NanoVan (Nanoprobes, Yaphank, NY, USA) and observed using a Jeol JEM 1010 electron microscope (Jeol, Tokyo, Japan) as previously described [26].

### Hepatocellular Function

Blood samples were collected by cardiac puncture; serum was then separated by centrifugation (10 min at 1200 g) and stored at −80°C. Serum levels of aspartate amino-transferase (AST), alanine amino-transferase (ALT) and lactate dehydrogenase (LDH) were assessed by standard absorption techniques at the biochemistry laboratory (Baldi e Riberi – Molinette Hospital).

### IVIS Analysis

Biodistribution analyses were performed using IVIS 200 small animal imaging system (Perkin Elmer, Waltham, MA, USA). After six hours, mice were sacrificed and organs collected. Hence, liver, kidneys, heart, lungs, spleen, pancreas were placed in a non-fluorescent Petri dish and the filters were set at 640 nm (Ex) and 700 nm (Em). Images were acquired and analyzed using Living Image 4.0 software (Perkin Elmer) through the designation of regions-of-interest. The fluorescence intensity was obtained and expressed as the Average Radiant Efficiency ([p/s/cm^2^/sr] / [μW/cm^2^]).

### Histological Analysis

Tissue biopsies collected from the ischemic lobes were formalin fixed and paraffin embedded for hematoxylin-eosin staining (H&E). The severity of histological damage was blindly scored by an experienced liver pathologist (E.D.) using the Suzuki’s Score: according to this system, congestion, ballooning degeneration and necrosis are graded from 0 to 4 [27].

HLSC-EV uptake was analyzed by immunofluorescence microscopy. After rinsing in PBS, slices were incubated for 5 min at 4°C with a permeabilization solution containing 20 mmol/l Hepes, 50 mmol/L NaCL, 300 mmol/L sucrose, 3 mmol/L MagCl2, 0,5% Triton X- 100, pH 7.4. After washing with PBS, slices were incubated for 1 h at room temperature with a blocking solution of PBS added with 3% bovine albumin (both from Sigma-Aldrich) and incubated overnight at 4°C with an anti-mouse cytokeratin-8 primary antibody (1:200) (Abcam). At the end of the incubation, they were washed with PBS and then incubated for 1 h at room temperature with the Alexa Fluor 488-conjugated secondary antibody (1:200) (Invitrogen). Thereby, slices were washed with PBS and nuclei were stained with Hoechst. After a final washing in PBS, slides were mounted with Fluoromount (Sigma-Aldrich). Microscopy analysis was performed using a Cell Observer SD-ApoTome laser scanning system (Carl Zeiss).

### Quantitative Reverse Transcription Polymerase Chain Reaction

Mouse hepatic tissue was suspended in 1 ml of TRIzol™ solution (Ambion,Thermofisher) and homogenized in a Bullet blender (Next Advance Inc., New York, NY, USA) at a speed of 8 rpm for 3 min using 0.5 mm size zirconium beads. The homogenized tissue was collected and centrifuged at 12,000 g for 15 min at 4°C, and the supernatant from homogenized tissue was transferred to clean tubes and subjected to RNA according to manufacturer’s protocol. Isolated RNA was quantified spectrophotometrically using NanoDrop 2000 (ThermoFisher Scientific). High-capacity cDNA reverse transcription Kit (Applied Biosystems™) was used to synthesize the cDNA from 200 ng of RNA. Then, a real-time polymerase chain reaction (RT-PCR) (Applied Biosystems™) was performed on duplicate cDNA samples according to the chemistry of Power SYBR® Green PCR Master Mix (Applied Biosystems™), using the primers described in supplementary material 2. Comparative ΔΔCt method was used to calculate the relative expression levels of the genes of interest normalized to the house-keeping gene expression Actinβ. Samples from the experimental sham group were used as reference for the quantitative analysis.

### Statistical Analysis

Data are expressed as mean ± standard error of the mean (SEM). Statistical analyses were performed using one-way ANOVA with Newman-Keuls multiple comparison test where appropriate (GraphPad Prism, version 6.00, USA). A *p* value <0.05 was considered as statistically significant.

## Results

### Characterization of HLSC-EV

**Fig. 1:**
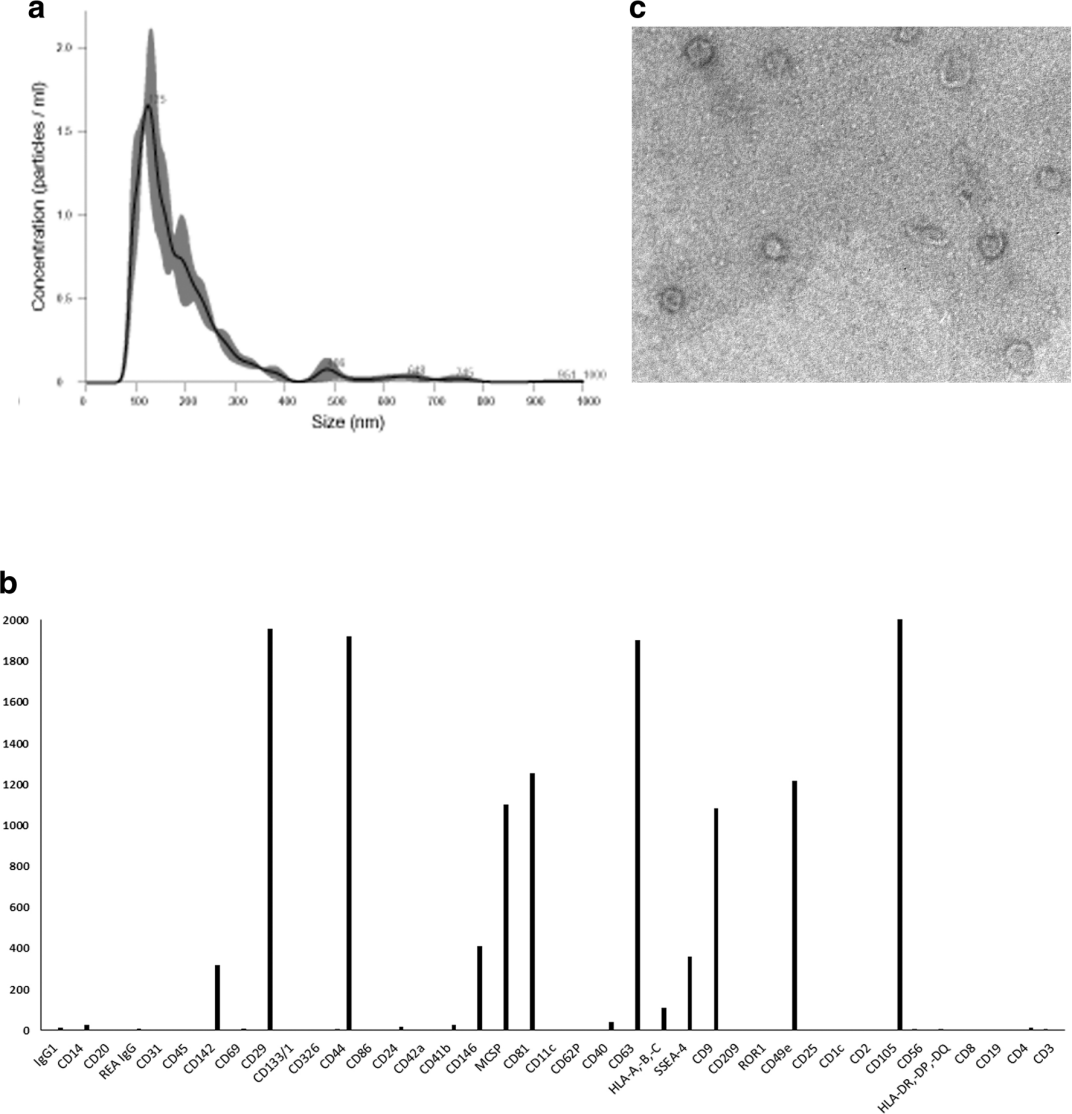
**Characterization of HLSC-EV.** (**a**) Nanoparticle tracking analyses showing the size distribution of purified HLSC-EV. (**b**) Cytofluorimetric characterization of HLSC-EV by multiplex bead-based flow cytometry assay: 39 multiplexed populations of dye-labeled antibody-coated capture beads are incubated with HLSC-EV samples. Captured HLSC-EV were counterstained with pan tetraspanins APC-labeled detection antibodies. The graph shows a quantification of the median APC fluorescence values for all bead populations after background correction (medium control values subtracted from measured HLSC-EV values) of a representative HLSC-EV preparation. (**c**) Representative micrograph of transmission electron microscopy of HLSC-EV. EV negatively stained with NanoVan (scale bars = 100 nm, magnification ×100,000)

Figure 1a reports the NanoSight profile of purified HLSC-EV. The expression of HLSC- EV markers was performed using the multiplex bead-based flow cytometry assay platform for EV as previously described [24, 25]. HLSC-EV expressed CD9, CD63, and CD81 as well as high fluorescence intensity for CD29, CD44, CD105 and CD49e. At low-positive fluorescence intensity were also detected other markers such as CD142, CD146, SSEA-4, and MCSP (Fig. 1b). Hematopoietic (CD3, CD4, CD8, CD19, etc.), endothelial (CD31), and epithelial (CD326) markers were negative in HLSC-EV. Moreover, HLSC-EV showed a homogeneous pattern of nano-sized membrane vesicles as seen by transmission electron microscopy (Fig. 1c).

### HLSC-EV Biodistribution

**Fig. 2:**
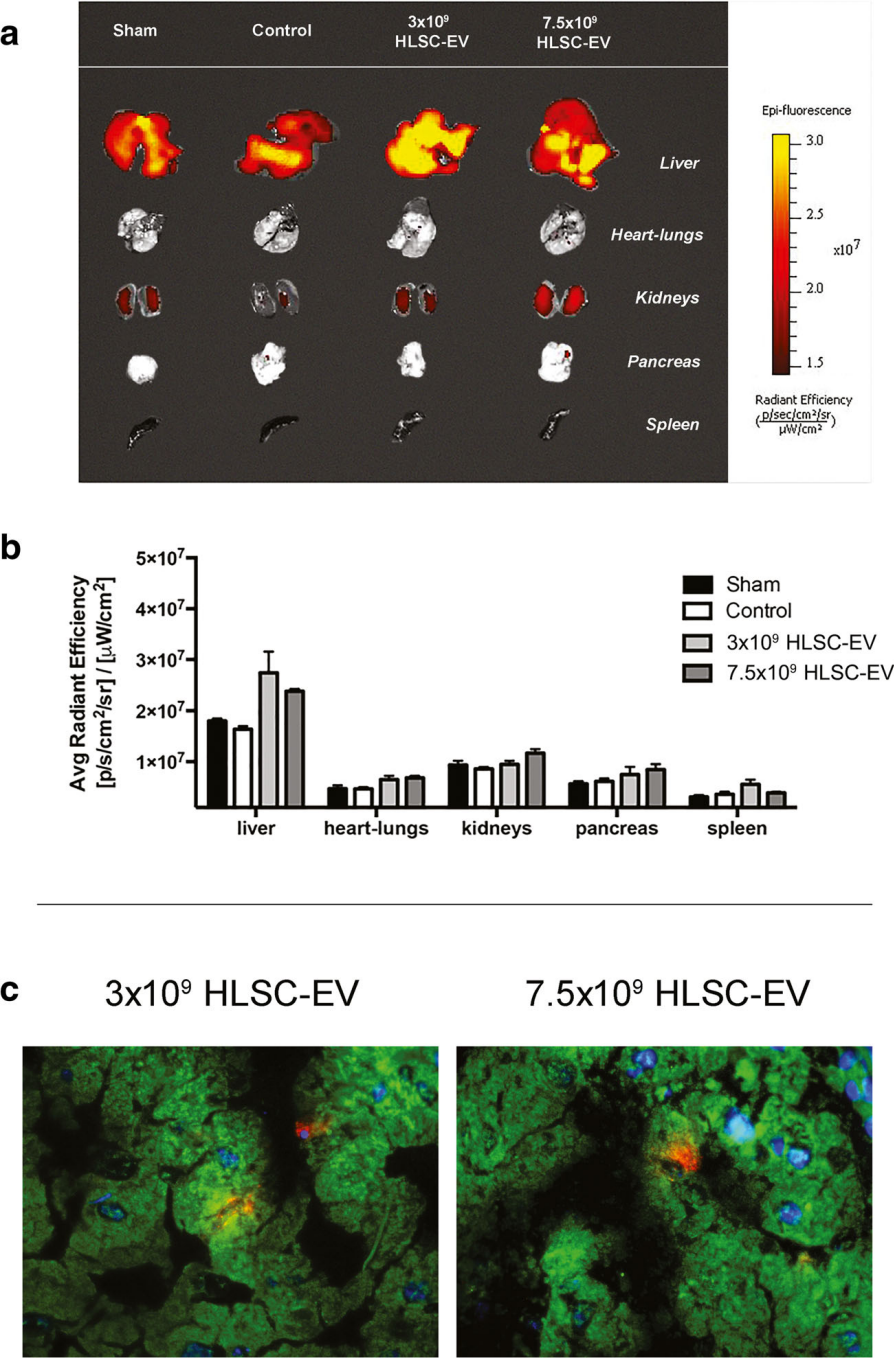
**Biodistribution and immunofluorescence of Dil/Did-stained HLSC-EV.** (**a**) Liver, heart and lungs, kidneys, pancreas and spleen accumulation of Dil/Did-stained HLSC-EV. Livers from control and sham operated animals exhibit increased fluorescence compared to other organs due to the characteristic strong background fluorescent signal of the liver. (**b**) Intensity of fluorescent signal detected ex-vivo after 6 h. In all groups, liver vs other organs (*p* < 0.0001), EV1 liver vs control liver (p < 0.0001) and EV1 liver vs EV2 liver (*p* < 0.01). Data are represented as mean ± SEM. (**c**) Representative micrographs showing DAPI-stained cell nuclei (blue), mouse anti-human cytokeratin-8 antibody immunofluorescence (green) and Dil/Did-stained HLSC-EV (red) (original magnification 630×)

The fluorescence from dissected organs was quantified immediately with IVIS (Fig. 2a). In all study groups, the fluorescent signal was significantly higher in the liver compared to other organs (Fig. 2b). The biodistribution analysis demonstrated that Dil-Did stained HLSC-EV were mostly localized in the liver but a modest signal was also detected in the kidneys, intestine, pancreas, spleen, heart and lungs. In fact, the hepatic fluorescence in the treated groups was significantly higher than the hepatic fluorescence in the control group, that received saline solution alone, and in the sham operated group (all *p* values <0.0001) (Fig. 2b). Finally, the livers of animals treated with the EV1 dose were significantly more fluorescent than the livers of mice treated with the EV2 dose (*p* < 0.05) (Fig. 2b). A relative increase, even if not significant, in the IVIS signal was also observed in kidneys of mice treated with the EV2 (Figs. 2a and b).

Furthermore, the presence of Dil-Did stained HLSC-EV was revealed by immunofluorescence analysis. In particular, the HLSC-EV were able to integrate within the hepatocytes, as confirmed by colocalization with the cytokeratin-8 antibody, used as a hepatocyte marker (Fig. 2c).

### Histological Analysis

**Fig. 3:**
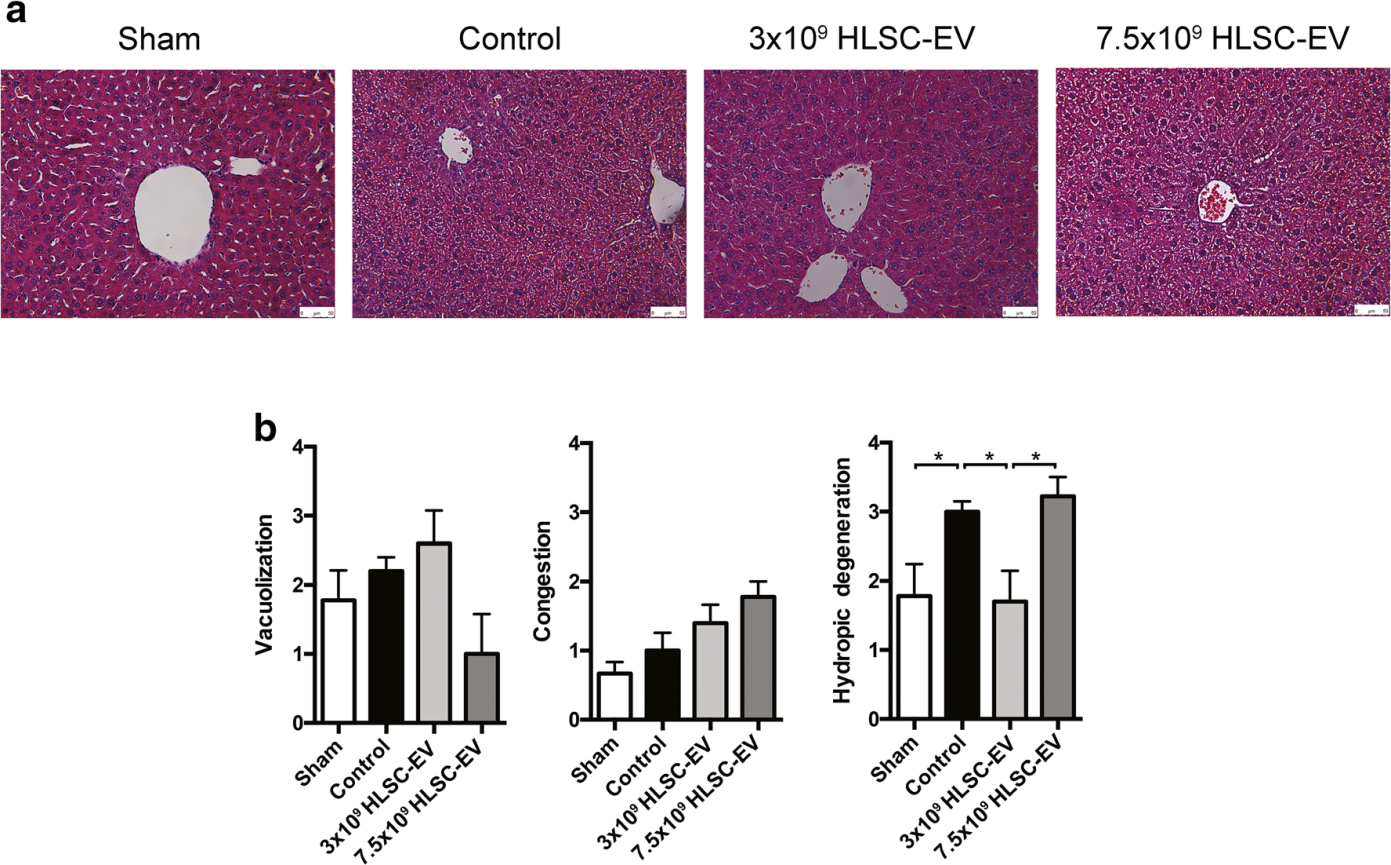
**Histological analysis showing the hepatoprotective activity of HLSC-EV against liver IRI.** (**a**) Representative micrographs of H&E stain of liver tissues (original magnification 200×, scale bar 50 μm). (**b**) Quantitative scoring for tissue damage according to Suzuki’s histological criteria (*p < 0.05). Data are represented as mean ± SEM

Compared with sham livers, large areas of vascular congestion, cell vacuolization and hydropic degeneration were observed in IRI mice (Fig. 3a). The Suzuki’s score quantification showed that the EV1 dose was able to reduce tissue damage, whereas this protective effect was not observed in the livers from the EV2 group, that were equal to controls. Since the animals were sacrificed after 6 h from IRI, hydropic degeneration was considered as a precursor of tissue necrosis that occurs in a longer period of time. In particular, the hydropic degeneration was significantly reduced in the EV1 group compared to control and EV2 group (EV1: 1.8 ± 0.37, control: 2.8 ± 0.17, EV2: 3.33 ± 0.17; *p* < 0.05), whereas vascular congestion and cell vacuolization were not modified (Fig. 3b). By contrast, livers from mice treated with the EV2 dose showed an increase in vascular congestion compared with those from sham mice (EV2: 1.78 ± 0.29, sham: 0.61 ± 0.14; p < 0.05) (Figs. 3a and b).

### Biochemistry Analysis

**Fig. 4:**
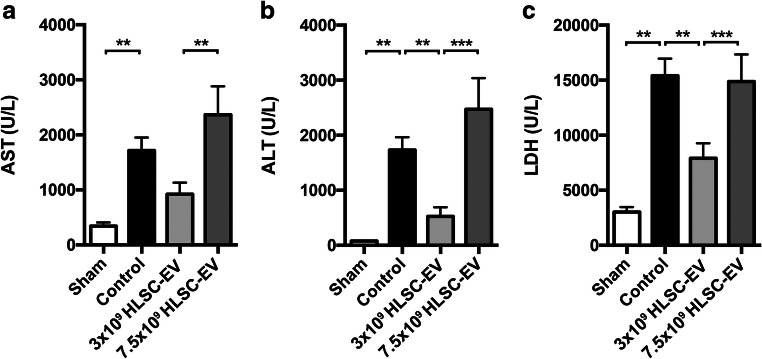
**Biochemical markers of liver injury showing the hepatoprotective activity of HLSC-EV against liver IRI.** (**a**) Aspartate aminotransferase (**p < 0.01) (**b**) Alanine aminotransferase (**p < 0.01, ***p < 0.001) and (**c**) Lactate dehydrogenase (**p < 0.01, ***p < 0.001). Data are represented as mean ± SEM

Six hours after reperfusion, serum levels of AST (control: 923 ± 210, sham: 343 ± 66.7 UI/L), ALT (control: 1733 ± 233, sham: 79.3 ± 10.6 UI/L) and LDH (control: 15414 ± 1552, sham: 3026 ± 443 UI/L) were significantly increased in the control group compared to the sham group (*p* < 0.01) (Figs. 4a, b and c). ALT and LDH levels were reduced in EV1 group compared to the control group (ALT EV1: 524 ± 168, control: 1733 ± 233 UI/L; LDH EV1 7905 ± 1374, control: 15414 ± 1552 UI/L; all *p* values<0.01), whereas this reduction was not observed for the EV2 group, that did not differ from controls (Figs. 4b and c).

### Molecular Biology

**Fig. 5:**
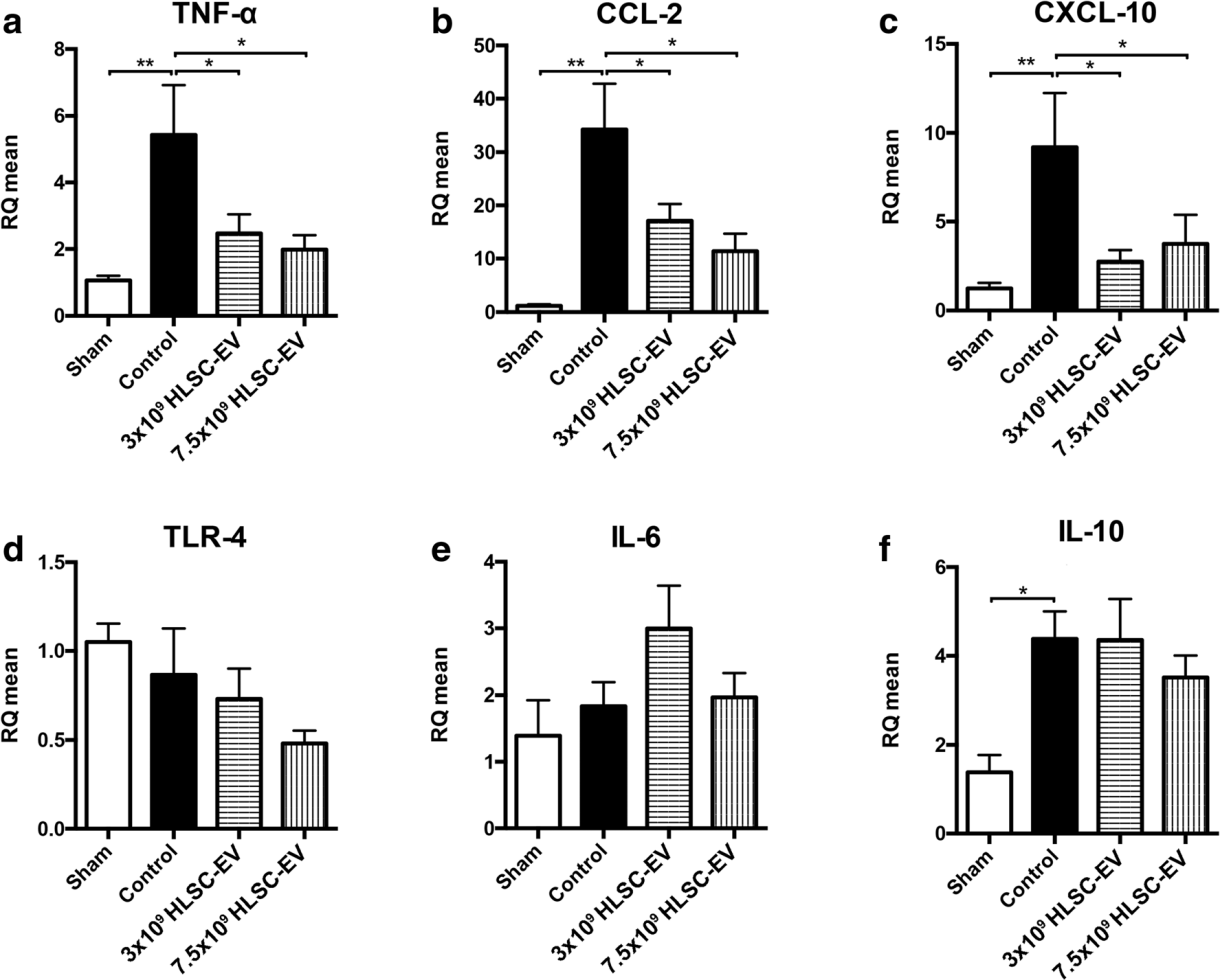
**Quantitative analysis of Real Time PCR on a selection of mouse genes involved in inflammation pathway.** Mean relative quantification of RT-PCR analysis of (**a**) TNF-α, (**b**) CCL-2, (**c**) CXCL-10, (**d**) TLR-4, (**e**) IL-6, and (**f**) IL-10. (*p < 0.05, **p < 0.01). All values are normalized to Actin β. Data are represented as mean ± SEM

To investigate at a molecular level whether HLSC-EV ameliorate injured IRI livers, gene expression of factors involved in cellular damage pathways were analyzed by RT-PCR. The mRNA levels of inflammatory molecules such as tumor necrosis factor alpha (TNF-α), CXC motif chemokine 10 (CXCL-10), CC motif chemokine ligand 2 (CCL-2) and interleukin 10 (IL-10) were increased at 6 h after the IRI induction (*p* < 0.01) (Figs. 5a-c, f). Compared to controls, expression levels of these mRNA were significantly reduced in HLSC-EV-treated IRI mice (p < 0.05), independently of the used dose (Figs. 5a, b, c). Expression of toll-like receptor 4 (TLR-4) and interleukin 6 (IL-6) mRNA were not affected by IRI and were not modulated by HLSC-EV treatment (Figs. 5d and e). Mean IL-6 mRNA level was higher in EV1 group, but this finding did not reach statistical significance (Fig. 5e).

We also evaluated mRNA expression of BCL-2-associated X protein (BAX) (pro-apoptotic; supplementary material 3A), B cell lymphoma 2 (BCL-2) (anti-apoptotic; supplementary material 3B), heme oxygenase 1 (HO-1) and sirtuin 1 (SIRT1) proteins (anti-oxidant and cytoprotective molecules; supplementary material 4A and B), hypoxia-inducible factor-1α (HIF-1α; supplementary material 5A), metalloproteinase inhibitor 1 precursor (TIMP1) and transforming growth factor-β1 (TGF-β1) proteins (pro-fibrotic; supplementary material 5B and C). RT-PCR analysis showed that only HO-1 mRNA levels exhibit a trend of increment in the IRI livers (supplementary material 4A), whereas TGF-β1 mRNA level (supplementary material 5C) was reduced in IRI group (p < 0.01). No modifications were observed in other analyzed mRNA (supplementary material 3A-B, 4B and 5A-C).

## Discussion

Hepatic IRI can be observed in different clinical settings such as liver resection and liver transplantation. The severity of IRI is proportional to the duration of the ischemic phase, which is characterized by anaerobic metabolism, accumulation of lactates and acidification of the extracellular milieu [2, 28, 29]. The restoration of oxygen and nutrients supply at reperfusion determines a sudden increase of ROS production, leading to mitochondria and cell damage, and subsequent activation of neutrophils and Kupffer cells [1, 30–34], which in turn release pro-inflammatory chemokines and cytokines [35–39]. Consequently, hepatocytes death can occur by apoptosis and oncotic necrosis [40, 41].

With regard to the liver setting, HLSC-EV successfully promoted liver regeneration in a model of 70% hepatectomy in rats [20]. Moreover, we recently demonstrated that HLSC-EV were able to reduce liver injury in a model of hypoxic normothermic machine perfusion. In this setting, we observed that HLSC-EV treatment significantly decreased AST and LDH release in the perfusate, necrosis and apoptosis severity and HIF-1α and TGF-β1 expression [21]. Recently, in a mouse model of NASH, HLSC-EV-treated animals exhibited reduced fibrosis and inflammation and proteins carried by HLSC-EV were identified as possible mediators of these effects [19].

HLSC-EV can be stored for up to 6 months at −80°C without losing their biological activity, which represents a relevant advantage of their use over that of stem cells.

On this basis, our study aimed at evaluating the ability of HLSC-EV to protect the liver against warm IRI in an in vivo mouse model. For this purpose, we used a well described mouse model of hepatic IRI [42]. The duration of the ischemic phase was set at 90 min, as this is the reported limit for hepatocyte survival in murine models [43]. Immediately after clamp removal, HLSC-EV treatment was administered systemically by the tail vein. The two doses studied were defined according to our laboratory experience on the use of HLSC-EV in vivo, [8, 19, 44] and other authors’ studies. All the animals were comparable in size and weight, resulting in low variability between the groups during surgical procedures. The biodistribution study confirmed the ability of HLSC-EV to localize within the hepatocytes of the damaged liver [21]. IVIS analysis showed that hepatic fluorescence was higher than that of other organs in all treatment groups including the sham operated group suggesting that liver is the organ mainly involved in the clearance of EV from circulation. However, signal from the EV1 livers was significantly higher than the sham, control and EV2 livers, suggesting that in this group HLSC-EV were effectively integrated in the hepatic parenchyma. The reason for the lower liver localization of the higher dose of EV is unclear but correlate with the reduced protective effect of EV2 on IRI.

The release of cytolytic enzymes is widely considered as an important marker of liver injury in mouse models of hepatic IRI [42, 45–47]. The EV1 dose significantly reduced serum ALT and LDH when compared to the control group. Furthermore, EV1 group did not differ from the sham group in terms of ALT release, showing that HLSC-EV treatment strongly protected the hepatocytes from the ischemic insult. On the other hand, there was no difference in ALT and LDH levels between EV2 and control group, suggesting that a higher EV dose failed to protect the liver against IRI.

Histological analyses were consistent with biochemistry results. In particular, only EV1 dose was able to reduce the amount of hydropic degeneration as compared to the control group, confirming the role of the lower dose of HLSC-EV in limiting IRI [21]. These results suggest that when EV are administered at higher concentrations they lose the majority of their beneficial role. This unexpected effect may be explained by the lower hepatic concentration reached by the EV2 dose, or because at this higher dose they exert a procoagulant activity, that was demonstrated also by other researchers [48]. On the other hand, excluding the liver and a tendency in the kidneys, there were no differences in biodistribution between the two doses and no intrahepatic clotting was observed within the livers in our model. Thus, this aspect remains unclear and further investigations are warranted to better define this dose-response relationship.

In our study, HLSC-EV treatment resulted in a significant decrease of TNF-α, CCL-2 and CXCL-10 mRNA levels, which are key inflammatory molecules that participate in the post-reperfusion phase of IRI [35, 36, 38, 39]. The expression of TLR-4, IL-6 and IL-10 was not affected by HLSC-EV. Also, we noted that HO-1 and SIRT1 mRNA levels in the IRI group were not different from those observed in sham animals, suggesting the absence of oxidative damage at 6 h after reperfusion.

In this in vivo model, HLSC-EV treatment did not influence BAX and BCL-2 mRNA levels in the livers exposed to IRI, indicating a lack of modulating effects on apoptosis by HLSC-EV. Nevertheless, in EV1 group we observed a reduction in the degree of hydropic degeneration, a precursor of necrosis, which could therefore represent the main process involved in hepatocytes loss in our model [49]. Finally, hypoxia did not lead to an early activation of fibrosis pathways in our experiments, as HIF1-α and TGF-β1 mRNA levels were similar between sham and ischemic groups. Moreover, we observed that TIMP1, which is activated downstream in the fibrotic process initiated by TGF-β1, was not activated, due to the lack of TGF-β1 activation.

Overall, our data suggest that the HLSC-EV are able reduce liver IRI by modulating the inflammatory status which characterizes the early phases of IRI, by acting at the beginning of the inflammatory cascade. Indeed, TNF-α is involved in the activation of chemokines cascade and it is produced by activated macrophages, CD4^+^ lymphocytes, neutrophils, mast cells and eosinophils, whereas CXCL-10 and CCL-2 are mainly secreted by Kupffer cells during hepatic inflammation, promoting neutrophils attraction [50, 51]. In our study, HLSC-EV reduced the production of TNF-α and, as a consequence, the production of the two other chemokines CXCL-10 and CCL-2, which are located downstream in the activation of the inflammatory cascade, thus ameliorating the local inflammation induced by the ischemia-reperfusion damage. Interestingly, also the EV2 dose was able to reduce the expression of TNF-α, CCL-2 and CXCL-10 genes, but this beneficial effect was observed only at molecular level and was not supported by biochemistry and histology results.

We observed that, after six hours of reperfusion, some key genes involved in inflammation were upregulated by IRI, and some of these genes were also modulated by HLSC-EV. This beneficial effect was also demonstrated by the reduction of cytolysis markers and hydropic degeneration in the animals treated with the lower dose of HLSC-EV. However, we acknowledge that our preliminary study presents certain limitations. In particular, we focused our attention on the acute phase of liver IRI and long-term effects were not investigated at this time. Since in our previous experience we found that HLSC-EV exert their properties within the first hours from reperfusion, our intention was to better understand the biological and molecular mechanisms involved in their early activity. It would be interesting in future studies to evaluate harder clinical outcomes, such as longer follow-up times and survival analyses.

In conclusion, this study demonstrates that a dose of 3 × 10^9^ HLSC-EV was able to protect the liver from IRI, whereas a dose of 7.5 × 10^9^ HLSC-EV was ineffective in ameliorating liver function, with only an anti-inflammatory modulation effect observed only at molecular level.

Altogether, these data may suggest that systemic administration of HLSC-EV could be considered as an alternative cell-based approach for hepatic ischemia reperfusion injury. Nevertheless, additional investigations are needed to further support the potential use of HLSC-EV in clinical settings.

## Supplementary Information

Supplementary material 1 **Selective clamping of intrahepatic pedicles.** The atraumatic clamp interrupted the blood flow to the left lateral and median lobes (black arrows), leading to an ischemia of approximately 70% of the hepatic parenchyma. The right and caudate lobes (white arrows) guaranteed a portocaval shunt that avoided intestinal congestion. (PNG 1512 kb)

High resolution image (TIF 57996 kb)

Supplementary material 2 **Primers’ sequences used for Real-Time PCR.** The forward and reverse sequences of primers used for RT-PCR to detect a selection of mouse genes are listed in the table. The selected mouse genes are: ACTβ (Actin β), BAX (BCL-2-associated X protein); BCL-2 (B cell lymphoma 2); CCL-2 (Chemokine C-C motif ligand 2); CXCL-10 (Chemokine C-X-C motif ligand 10); HIF-1α (Hypoxia inducible factor 1, alpha subunit); HO-1 (Heme oxygenase 1); IL-6 (Interleukin 6); IL-10 (Interleukin 10); SIRT1 (Sirtuin 1); TGF-β1 (Transforming growth factor β 1); TIMP1 (Tissue inhibitor of metalloproteinase 1); TLR-4 (Toll-like receptor 4); TNF-α (Tumor necrosis factor α). (DOCX 13 kb)

Supplementary material 3 **Quantitative analysis of RT-PCR on a selection of mouse genes involved in apoptosis pathway.** Mean relative quantification of RT-PCR analysis of (A) BAX and (B) BCL-2. All values are normalized to Actin β. Data are represented as mean ± SEM. (PNG 130 kb)

High resolution image (TIF 3332 kb)

Supplementary material 4 **Quantitative analysis of RT-PCR on a selection of mouse genes involved in oxidative stress pathway.** Mean relative quantification of RT- PCR analysis of (A) HO-1 and (B) SIRT1. All values are normalized to Actin β. Data are represented as mean ± SEM. (PNG 129 kb)

High resolution image (TIF 3392 kb)

Supplementary material 5 **Quantitative analysis of RT-PCR on a selection of mouse genes involved in hypoxia and fibrosis pathways.** Mean relative quantification of RT-PCR analysis of (A) HIF-1α, (B) TIMP1 and (C) TGF-β1. (**p < 0.01). All values are normalized to Actin β. Data are represented as mean ± SEM. (PNG 134 kb)

High resolution image (TIF 4984 kb)
