# Supplementary figures and images for: Protective Effects of Human Liver Stem Cell-Derived Extracellular Vesicles in a Mouse Model of Hepatic Ischemia-Reperfusion Injury
Source: Stem Cell Rev Rep. 2020 Dec 2;17(2):459–70. doi: 10.1007/s12015-020-10078-7 (PMC8036187; doi:10.1007/s12015-020-10078-7)

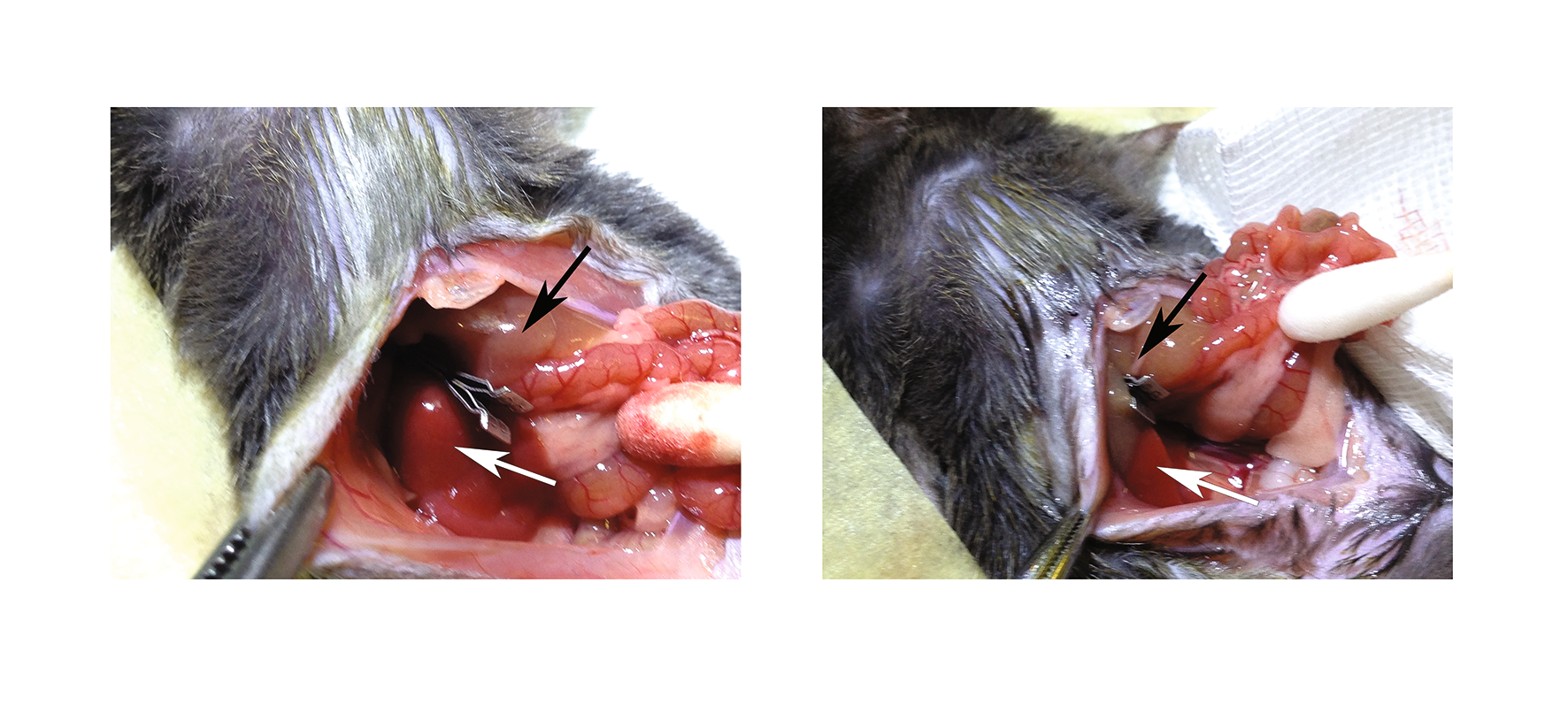

Supplement: Supplementary file 1 — Supplementary material 1 Selective clamping of intrahepatic pedicles. The atraumatic clamp interrupted the blood flow to the left lateral and median lobes (black arrows), leading to an ischemia of approximately 70% of the hepatic parenchyma. The right and caudate lobes (white arrows) guaranteed a portocaval shunt that avoided intestinal congestion. (PNG 1512 kb) [file 12015_2020_10078_Fig6_ESM.png]

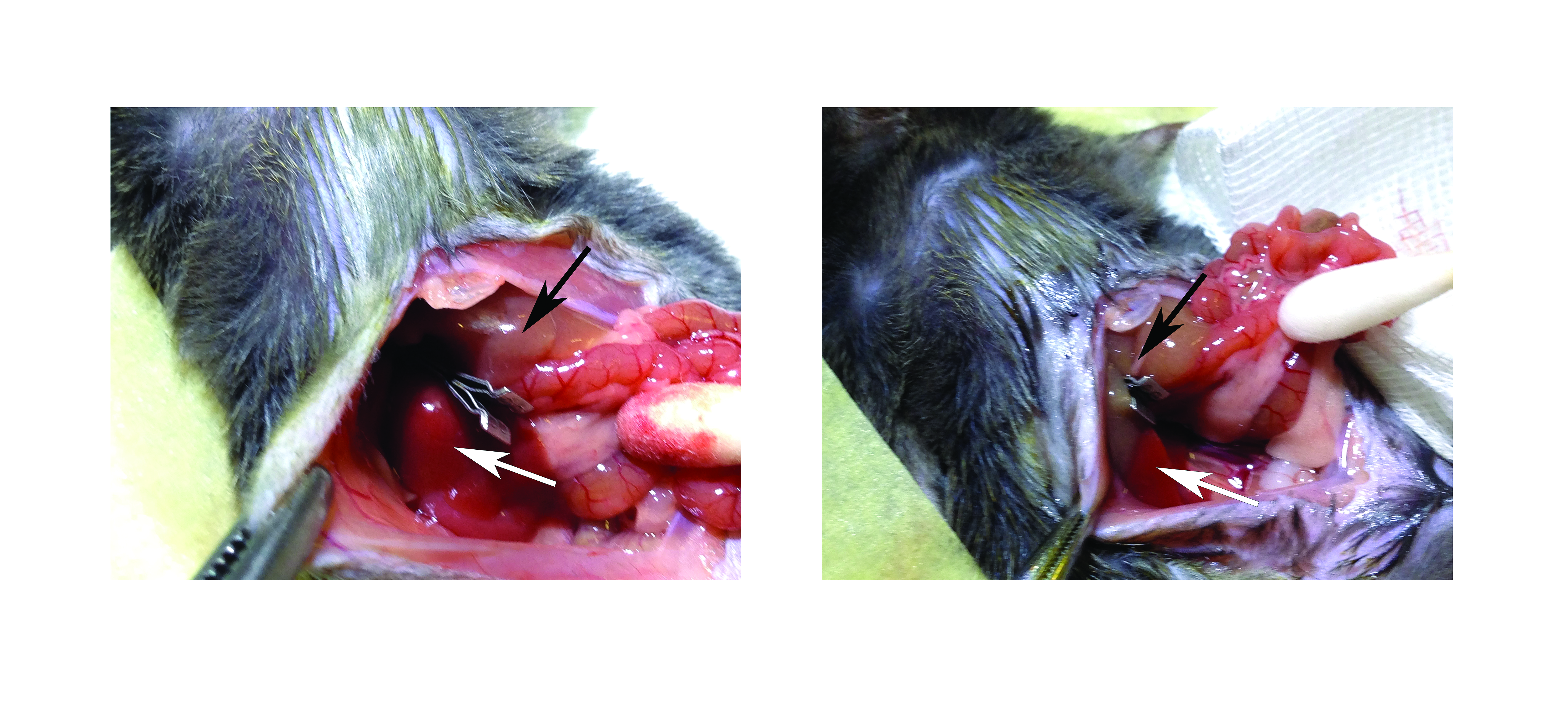

Supplement: Supplementary file 2 — High resolution image (TIF 57996 kb) [file 12015_2020_10078_MOESM1_ESM.tif]

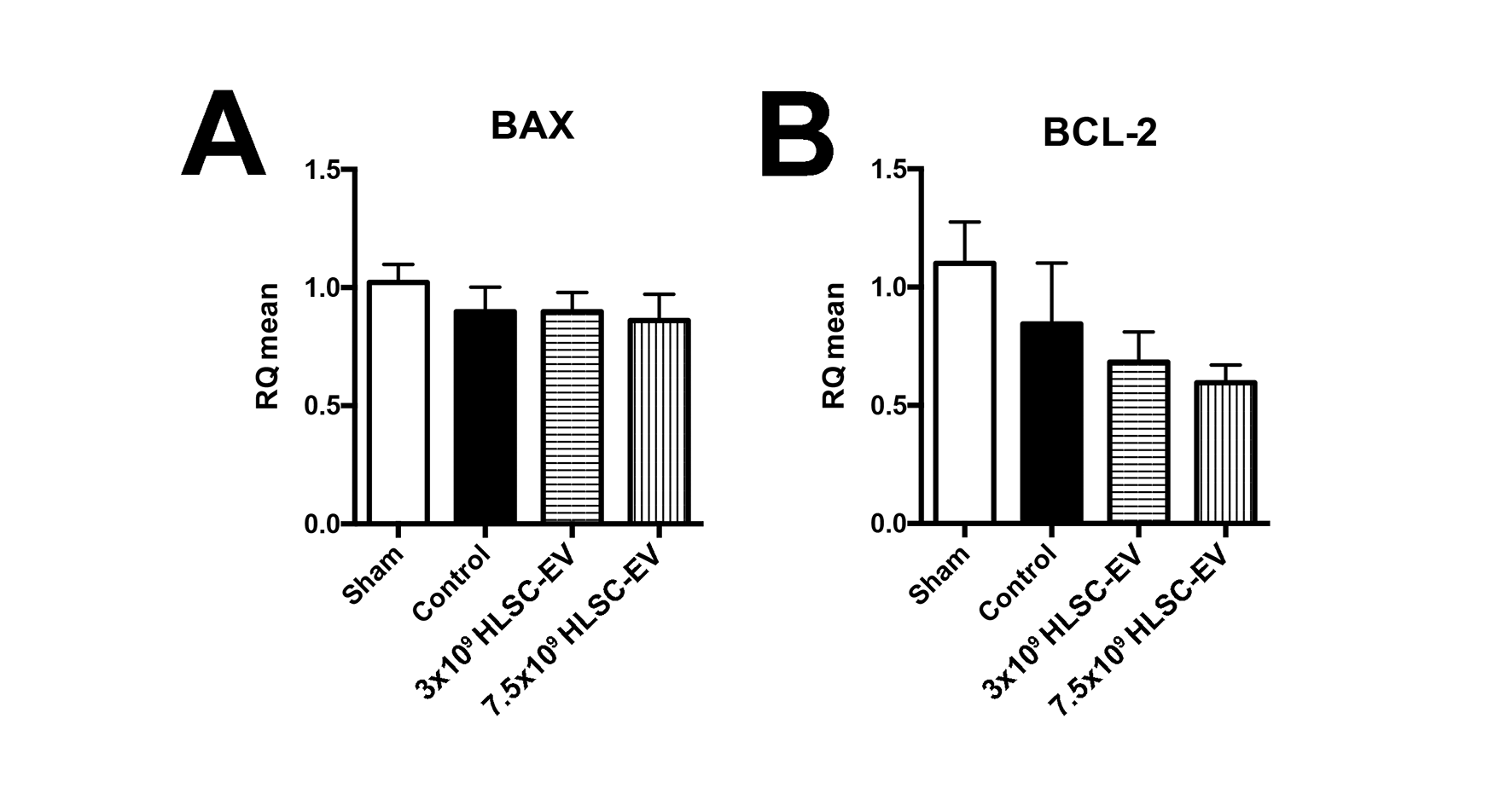

Supplement: Supplementary file 4 — Supplementary material 3 Quantitative analysis of RT-PCR on a selection of mouse genes involved in apoptosis pathway. Mean relative quantification of RT-PCR analysis of (A) BAX and (B) BCL-2. All values are normalized to Actin β. Data are represented as mean ± SEM. (PNG 130 kb) [file 12015_2020_10078_Fig7_ESM.png]

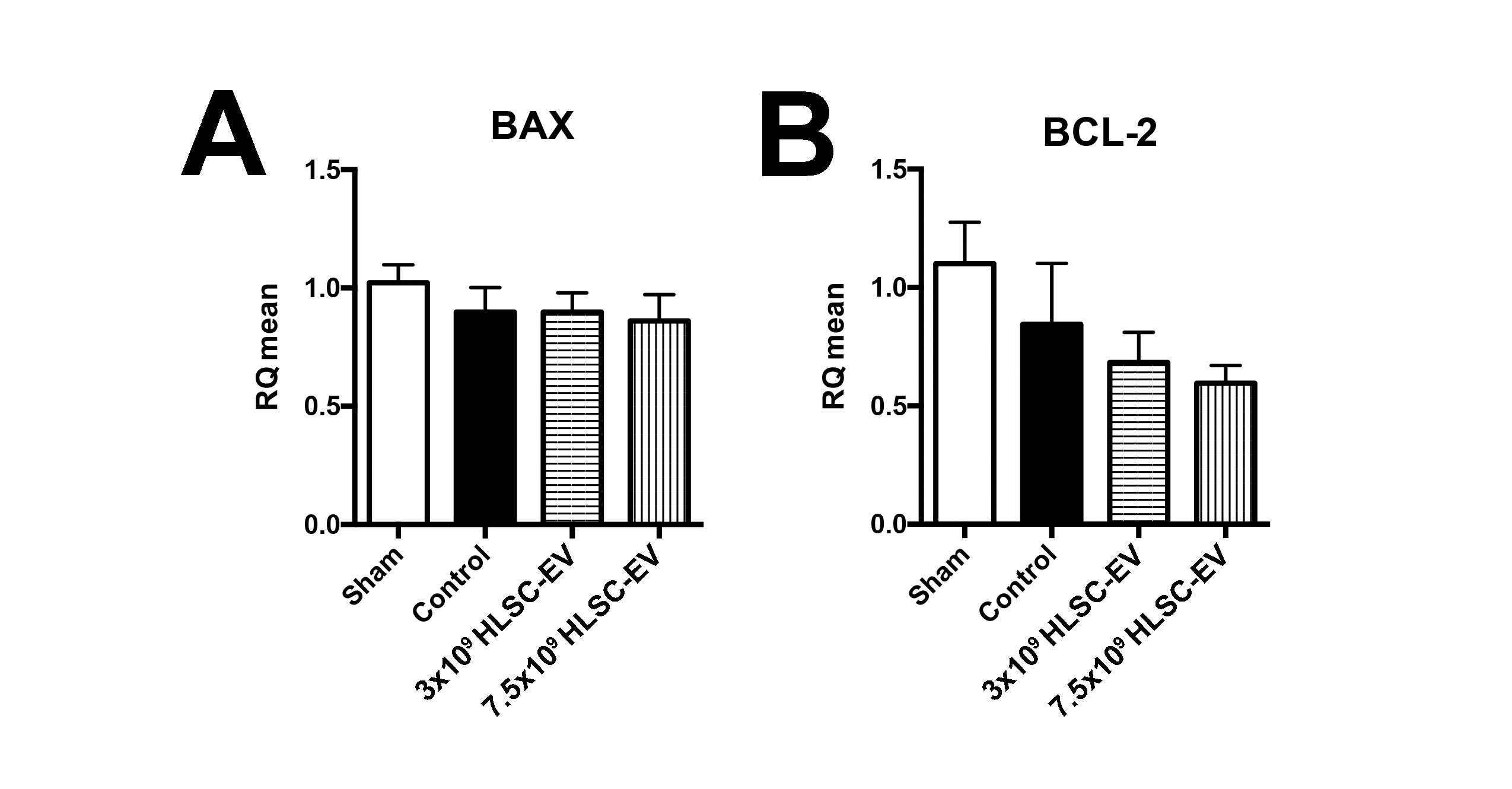

Supplement: Supplementary file 5 — High resolution image (TIF 3332 kb) [file 12015_2020_10078_MOESM3_ESM.tif]

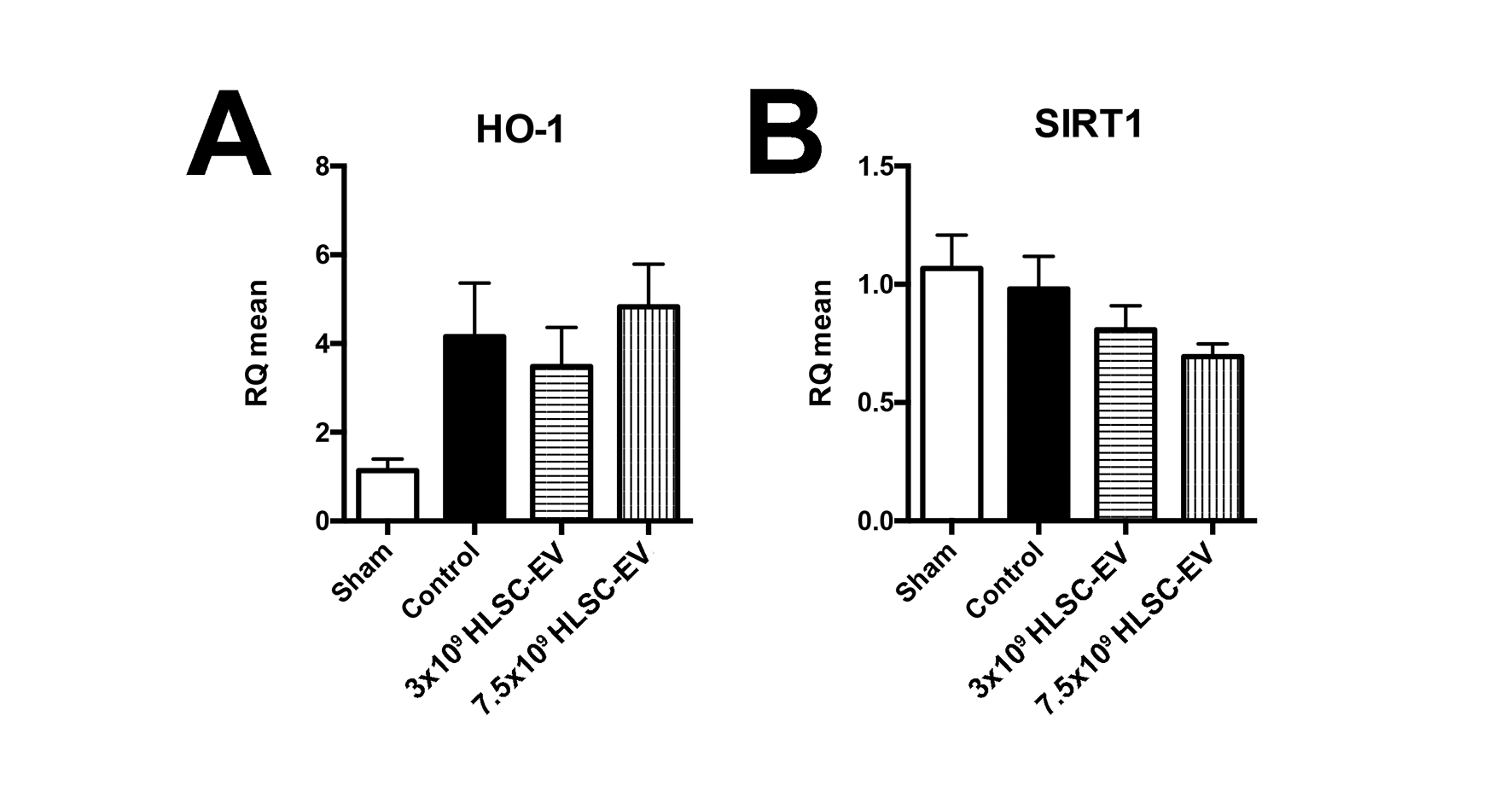

Supplement: Supplementary file 6 — Supplementary material 4 Quantitative analysis of RT-PCR on a selection of mouse genes involved in oxidative stress pathway. Mean relative quantification of RT- PCR analysis of (A) HO-1 and (B) SIRT1. All values are normalized to Actin β. Data are represented as mean ± SEM. (PNG 129 kb) [file 12015_2020_10078_Fig8_ESM.png]

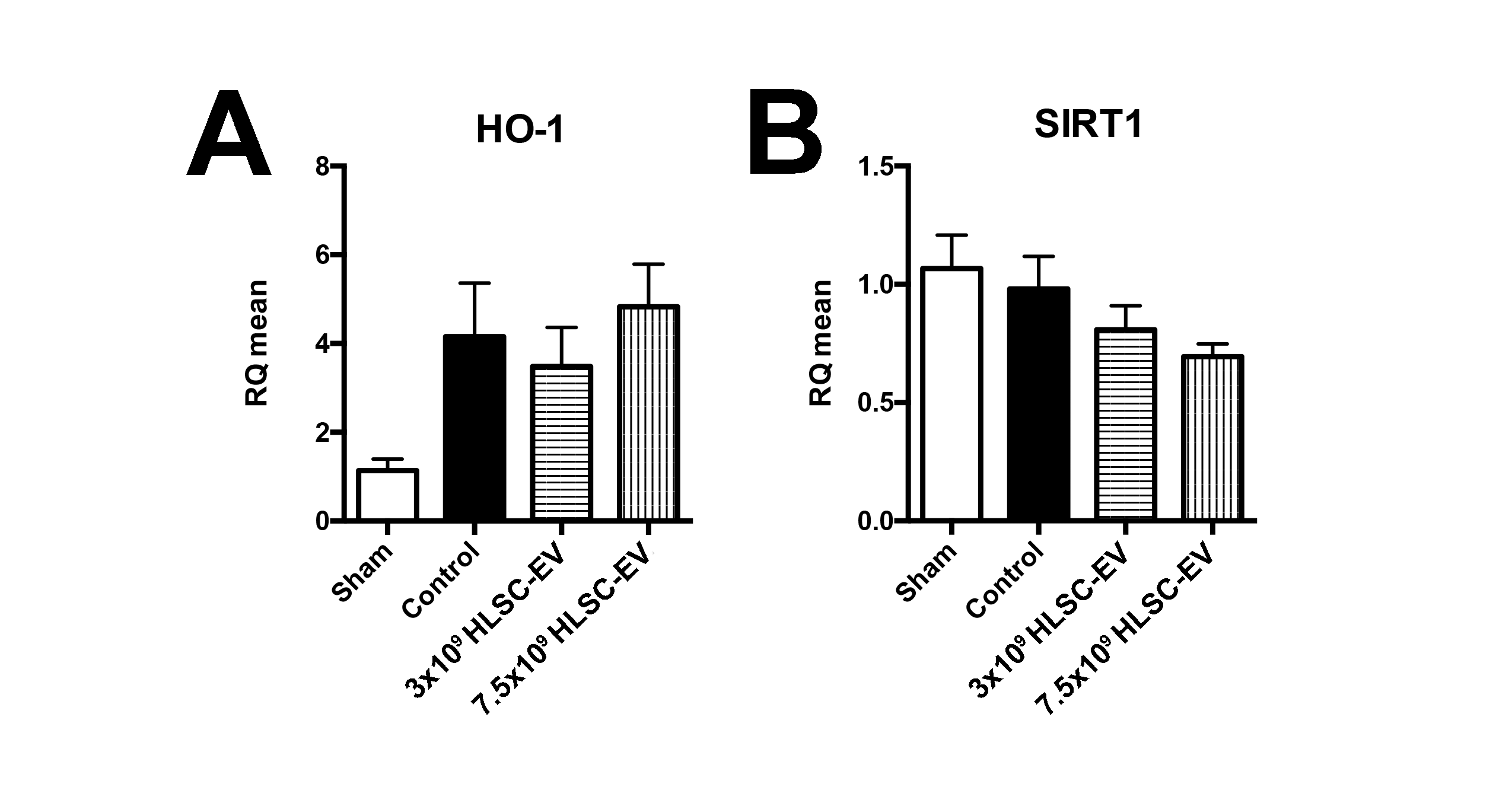

Supplement: Supplementary file 7 — High resolution image (TIF 3392 kb) [file 12015_2020_10078_MOESM4_ESM.tif]

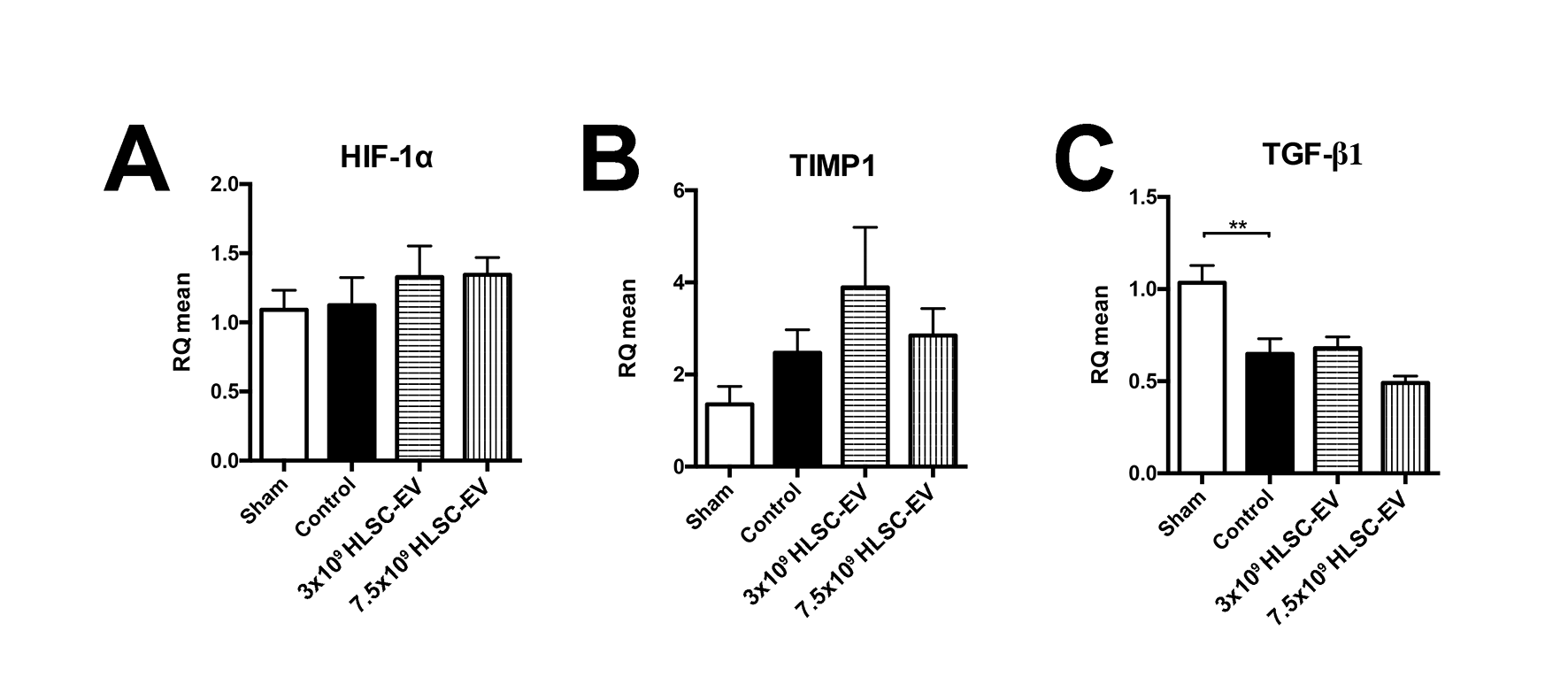

Supplement: Supplementary file 8 — Supplementary material 5 Quantitative analysis of RT-PCR on a selection of mouse genes involved in hypoxia and fibrosis pathways. Mean relative quantification of RT-PCR analysis of (A) HIF-1α, (B) TIMP1 and (C) TGF-β1. (**p < 0.01). All values are normalized to Actin β. Data are represented as mean ± SEM. (PNG 134 kb) [file 12015_2020_10078_Fig9_ESM.png]

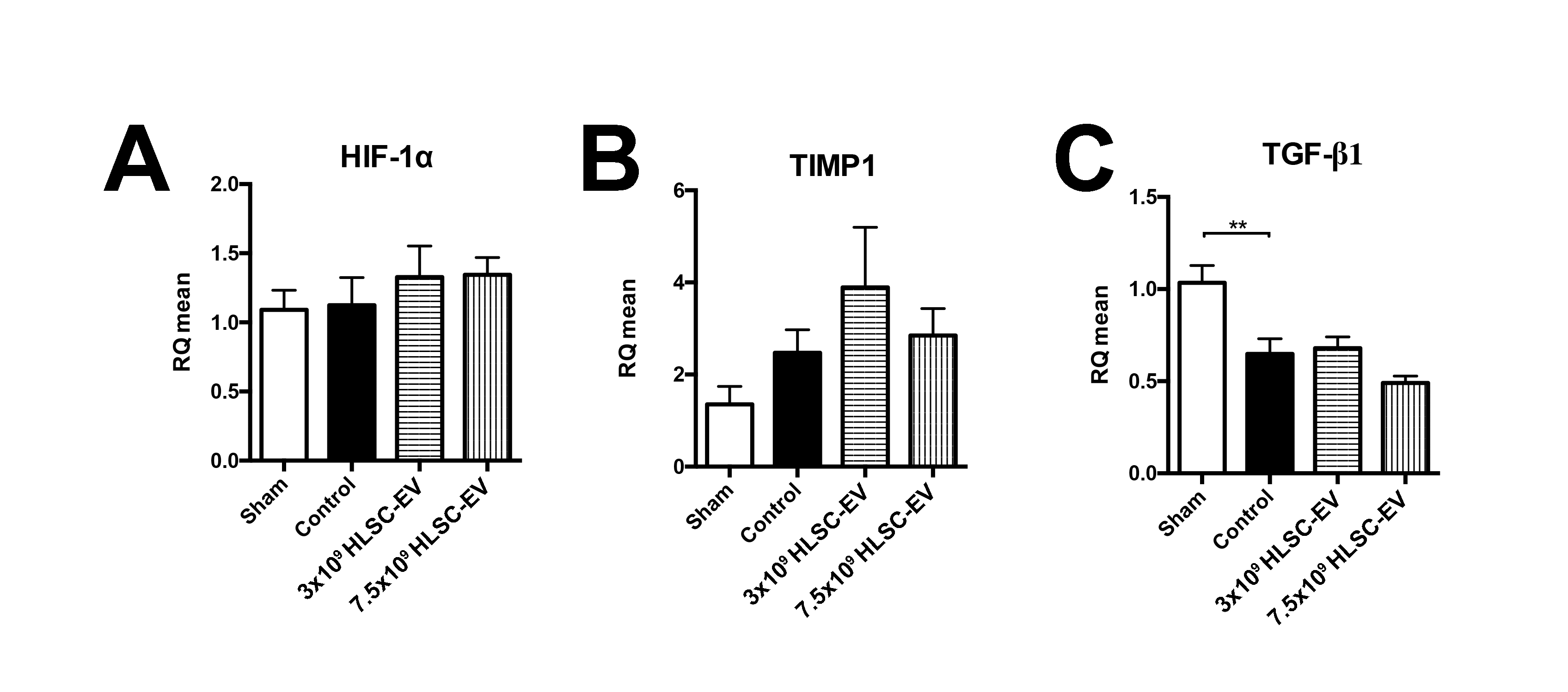

Supplement: Supplementary file 9 — High resolution image (TIF 4984 kb) [file 12015_2020_10078_MOESM5_ESM.tif]
