# Supplementary material for: Protective Effects of Human Liver Stem Cell-Derived Extracellular Vesicles in a Mouse Model of Hepatic Ischemia-Reperfusion Injury
Source: Stem Cell Rev Rep. 2020 Dec 2;17(2):459–70. doi: 10.1007/s12015-020-10078-7 (PMC8036187; doi:10.1007/s12015-020-10078-7)
Supplement: Supplementary file 3 — Supplementary material 2 Primers’ sequences used for Real-Time PCR. The forward and reverse sequences of primers used for RT-PCR to detect a selection of mouse genes are listed in the table. The selected mouse genes are: ACTβ (Actin β), BAX (BCL-2-associated X protein); BCL-2 (B cell lymphoma 2); CCL-2 (Chemokine C-C motif ligand 2); CXCL-10 (Chemokine C-X-C motif ligand 10); HIF-1α (Hypoxia inducible factor 1, alpha subunit); HO-1 (Heme oxygenase 1); IL-6 (Interleukin 6); IL-10 (Interleukin 10); SIRT1 (Sirtuin 1); TGF-β1 (Transforming growth factor β 1); TIMP1 (Tissue inhibitor of metalloproteinase 1); TLR-4 (Toll-like receptor 4); TNF-α (Tumor necrosis factor α). (DOCX 13 kb) [file 12015_2020_10078_MOESM2_ESM.docx]

**SUPPLEMENTARY MATERIAL 2: Primers’ sequences used for Real-Time PCR**

| **Gene** | **Forward primer sequence (5’→3’)** | **Reverse primer sequence (5’→3’)** |
| --- | --- | --- |
| BAX | TTTTGCTACAGGGTTTCATCCAG | GCCGGAGACACTCGCTCA |
| BCL-2 | GAGGCTGGGATGCCTTTGT | CCAGGTATGCACCCAGAGTGA |
| CCL2 | AAGCTGTAGTTTTTGTCACCAAGCT | TGGTTCCGATCCAGGTTTTTA |
| CXCL10 | GTGTTGAGATCATTGCCACGAT | GGCTAAACGCTTTCATTAAATTCTTG |
| HIF1α | TTCTGGATGCCGGTGGTC | GTCGCCGTCATCTGTTAGCA |
| HO1 | GTCGCCGTCATCTGTTAGCA | GTTGCCAACAGGAAGCTGAGA |
| IL-6 | AAGAGTTGTGCAATGGCAATTCT | TGATTATATCCAGTTTGGTAGCATCC |
| IL-10 | GACTTTAAGGGTTACTTGGGTTGC | TCCTGAGGGTCTTCAGCTTCTC |
| SIRT1 | CTACCGAGACAACCTCCTGTTGA | CCAGTCACTAGAGCTGGCGTG7 |
| TGFβ1 | CAACAACGCCATCTATGAGAAAAC | CTCTGCACGGGACAGCAAT |
| TIMP1 | GACCTGGTCATAAGGGCTAAATTC | TTAGTCATCTTGATCTTATAACGCTGGTA |
| TLR4 | GAATCCCTGCATAGAGGTAGTTCC | GAATCCCTGCATAGAGGTAGTTCC |
| TNFα | AGGGATGAGAAGTTCCCAAATG | GCTTGTCACTCGAATTTTGAGAAG |
| ACTβ | GATTACTGCTCTGGCTCCTAGCA | GCCACCGATCCACACAGAGT |
